# Effects on Fetal Metabolic Programming and Endocannabinoid System of a Normocaloric Diet during Pregnancy and Lactation of Female Mice with Pregestational Obesity

**DOI:** 10.3390/nu15163531

**Published:** 2023-08-11

**Authors:** Cynthia Barrera, Valeska Castillo, Rodrigo Valenzuela, Carina A. Valenzuela, Diego F. Garcia-Diaz, Miguel Llanos

**Affiliations:** 1 Department of Nutrition, Faculty of Medicine, University of Chile, Santiago 8380000, Chile; cbarrera@uchile.cl (C.B.); rvalenzuelab@uchile.cl (R.V.); digarcia@uchile.cl (D.F.G.-D.); 2 Laboratory of Nutrition and Metabolic Regulation, Institute of Nutrition and Food Technology (INTA), University of Chile, Santiago 8380453, Chile; valeska.castillo@inta.uchile.cl; 3 Eating Behavior Research Center, School of Nutrition and Dietetics, Faculty of Pharmacy, Universidad de Valparaíso Playa Ancha, Valparaíso 2360102, Chile; carina.valenzuela@uv.cl

**Keywords:** obesity, pregnancy, lactation, fetal programming, nutritional intervention, endocannabinoid system

## Abstract

Fetal programming provides explanatory mechanisms for the currently high prevalence of gestational obesity. The endocannabinoid system (ECS) participates in the regulation of energy balance, and with a high-fat diet (HFD), it is overactivated. The aim of this study was to determine the effects of a nutritional intervention during pregnancy and lactation on obese female progenitors, on metabolic alterations of the offspring and on the involvement of ECS. Female mice (C57/BL/6-F0), 45 days old, and their offspring (males) were separated according to type of diet before and during gestation and lactation: CON-F1: control diet; HFD-F1 group: HFD (fat: 60% Kcal); INT-F1 group: HFD until mating and control diet (fat: 10% Kcal) afterward. Glucose tolerance and insulin sensitivity (IS) were tested at 2 and 4 months. At 120 days, mice were sacrificed, plasma was extracted for the determination of hormones, and livers for gene expression and the protein level determination of ECS components. INT-F1 group presented a lower IS compared to CON-F1, and normal levels of adiponectin and corticosterone in relation to the HFD-F1 group. The intervention increased hepatic gene expression for fatty-acid amide hydrolase and monoacylglycerol lipase enzymes; however, these differences were not observed at the protein expression level. Our results suggest that this intervention model normalized some hormonal parameters and hepatic mRNA levels of ECS components that were altered in the offspring of progenitors with pre-pregnancy obesity.

## 1. Introduction

In different societies of the world, overnutrition is increasing in women of reproductive age. In the US, the country with the second highest prevalence of obesity in the OECD ranking [1], pre-pregnancy obesity increased from 26.1% in 2016 to 29.0% in 2019 for white women, non-Hispanic African Americans, and Hispanics [2]. Likewise, maternal obesity rates during pregnancy have increased significantly, and with it, the risk of developing obesity after the birth of the offspring [3]. Globally, 40 million children under 5 years of age were already overweight by 2018 [4]. Concerns about this situation, beyond the burden of disease that it entails, are directly related to the effects in the health of both the mother and the developing child, increasing the risk of complications in pregnancy such as gestational diabetes, preeclampsia, placental insufficiency, and maternal hypertension [5], as well as a high risk of macrosomic babies as a product [6]. The overfeeding of the mother during pregnancy and heavier babies at birth are considered risk factors related to the development of insulin resistance, obesity, and diabetes mellitus in postnatal life [7,8].

The adaptive process by which nutrition and other environmental factors alter developmental pathways during the prenatal growth period, thereby inducing changes in postnatal metabolism and adult susceptibility to chronic disease, is known as fetal programming [9]. Extensive epidemiological data show an association between maternal obesity and nutrition during pregnancy and the obesity of the offspring, and several animal models have been established to study the underlying mechanisms contributing to the programming of the systems [10]. In pregnant rodents, high-fat diets cause metabolic dysfunction in the adult offspring, but the mechanisms involved are not completely understood.

One of the mechanisms under study is related to the dysregulation of the endocannabinoid system (ECS). This system is mainly composed of cannabinoid receptors (cannabinoid receptor type 1 (RCB1) and cannabinoid receptor type 2 (RCB2)), their endogenous ligands called endocannabinoids, of which the best known are anandamide (AEA) and 2-arachidonoyl-glycerol (2-AG) [11], and their synthesis and degradation enzymes. Endocannabinoids are lipid compounds derived from arachidonic acid (ARA, 20:4n-6) and therefore, their biosynthesis can be influenced by the dietary fatty acids composition. After their release, both AEA and 2-AG are degraded by the enzyme fatty acid amido hydrolase (FAAH), and monoacylglycerol lipase (MAGL), respectively. CB1 receptors are expressed in the central nervous system and in several peripheral tissues that are related to the regulation of energy balance, such as liver, skeletal muscle, and adipose tissue [12]. Thus, the ECS plays an important role in lipogenesis, adipogenesis, and thermogenesis and early changes in offspring’s adipose stores [13]. A high dietary intake of linoleic acid (LNA, 18:2n-6), a precursor of ARA, is associated with a higher prevalence of obesity [14]. High-fat-diet-induced obesity is associated with fatty liver, insulin resistance, leptin resistance, and changes in the plasma lipid profile [15]. Endocannabinoids have been implicated in the development of these phenotypes, as CB1 cannabinoid receptor (CB1-/-) knockout mice do not develop the metabolic changes associated to obesity induced for diet [16]. Endocannabinoids regulate energy balance and lipid metabolism by stimulating the cannabinoid receptor type 1 (CB1) [17,18]. Genetic deletion and pharmacological antagonism have shown that CB1 signaling is necessary for the development of obesity and related metabolic disorders [16,19]. A gestational exposure to high-calorie diets generates an alteration of the ECS in the offspring, where its early activation results in an altered physiology of the tissues involved, developing characteristics of the metabolic syndrome that include increased adiposity, hyperleptinemia, hypertriglyceridemia, and hypercholesterolemia, even when pups were maintained on a control diet post-weaning [20].

**Figure 1:**
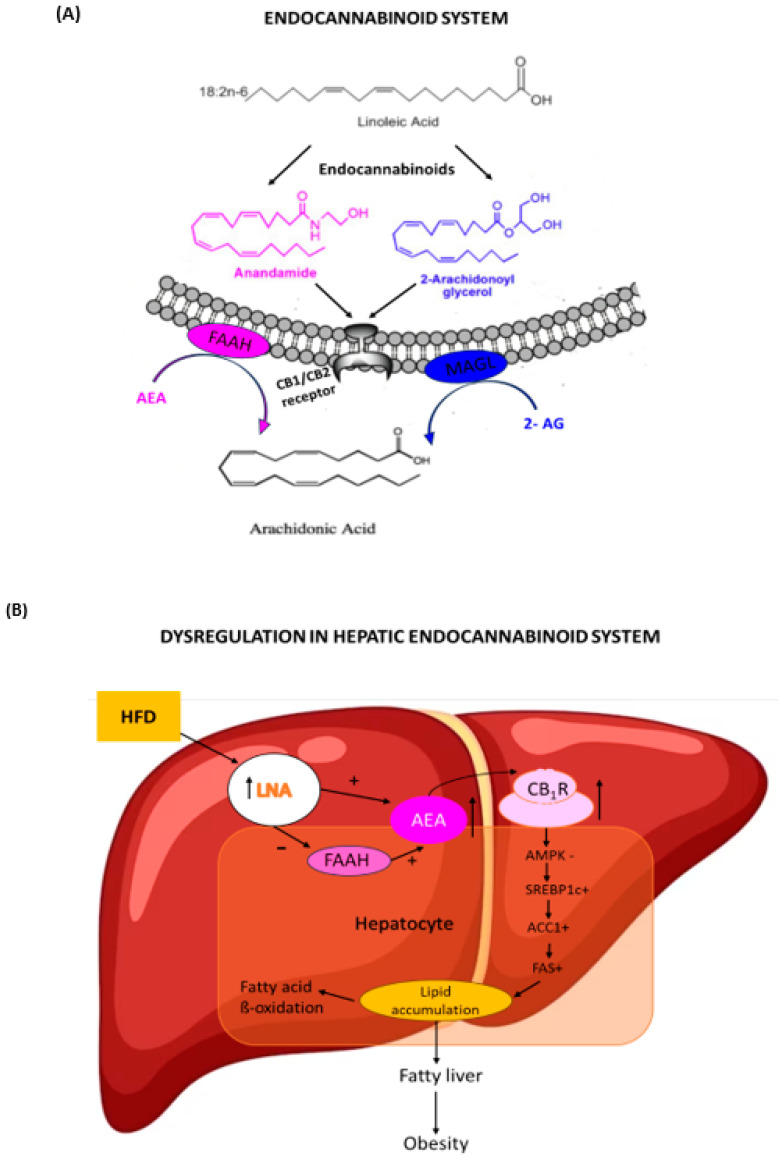
(**A**) Components of the endocannabinoid system. Endocannabinoids: anandamide (AEA), 2-arachidonoyl glycerol (2-AG); CB1 and CB2 receptors; endocannabinoid-degrading enzymes: fatty acid amide hydrolase (FAAH) and monoacylglycerol lipase (MAGL). (**B**) Schematic illustration of the dysregulation of the hepatic endocannabinoid system in response to a high-fat diet (HFD). LNA: Linoleic acid; AMPK: AMP-activated protein kinase; SREBP-1: sterol regulatory element-binding protein 1; ACC1: acetyl-CoA carboxylase; FAS: fatty acid synthase.

Although the literature widely describes the negative effects of excessive weight gain in pregnancy, nutritional intervention studies carried out during pregnancy show varied results and effectiveness [21,22,23]. Epidemiological and animal studies point to differences in offspring conceived during a time of maternal nutritional restriction. These include changes in hypothalamic–pituitary–adrenal axis function, body composition, glucose metabolism, and cardiovascular function [24]. In an animal model study, where mice fed a high-fat diet were switched to a control diet one week before pregnancy, showed that the diet change did not lead to weight loss of the female mice before pregnancy and there was no benefit for the offspring either, which developed obesity in adulthood [25]. However, it is still unclear if using a control diet during pregnancy and lactation in female mice with diet-induced obesity prevents the metabolic alterations in the adult offspring and if the ECS is implicated. Thus, the aim of this study was to determine the effect of a control diet during pregnancy and lactation, on the development of metabolic alterations, body composition and ECS activity in the adult offspring of obese mothers. Figure 1 shows the components of the endocannabinoid system and the effects of an overactive ECS in the liver that ultimately results in the development of obesity.

## 2. Materials and Methods

All treatment protocols for experimental animals were approved by the Bioethics Committee for Experimental Animals of the Institute of Nutrition and Food Technology, Universidad de Chile, Santiago, Chile (Protocol No. PT2016-03-CB-ML).

### 2.1. Experimental Animals

Fifteen male mice and 30 female C57BL/6J (Charles River) mice of 45 days old (estimated time in which this animal model presents sexual maturity) were acquired in the animal facility of Pontificia Universidad Católica de Chile. All the animals were kept in cages under standard humidity and temperature conditions (22–24 °C) during the experimentation period, with 12 h light/dark cycles and free access to purified water and food.

### 2.2. Experimental Design

The model considered three experimental groups, according to diet before and during pregnancy and lactation and their subsequent offspring (males only).

(a) CON-F0 group (control group): females with control diet before and during pregnancy and lactation (n = 5); and their offspring CON-F1 group (n = 10);
(b) HFD-F0 group (high fat diet group): females with HFD before and during pregnancy and lactation (n = 4); and their offspring HFD-F1 group (n = 10);
(c) INT-F0 group (intervention group): females with HFD until mating and control diet during pregnancy and lactation (intervention group, n = 6); and their offspring INT-F1 group (n = 10).

To obtain a group of normal-weight females and another group with obesity prior to mating, 30 female mice with similar body weights were randomly divided into two groups. At 45 days old, when they were acquired, they started a control diet (CON, n = 10) or a high-fat diet (HFD, n = 20), maintaining said diets for 10 weeks. At the end of this period, the mice fed with HFD that gained 25% more body weight, were considered overweight, compared to the average weight of the animals fed with a control diet. The male mice were always fed with a control diet, except on the mating days, when they were fed according to the diet assigned to the females (4 days).

**Figure 2:**
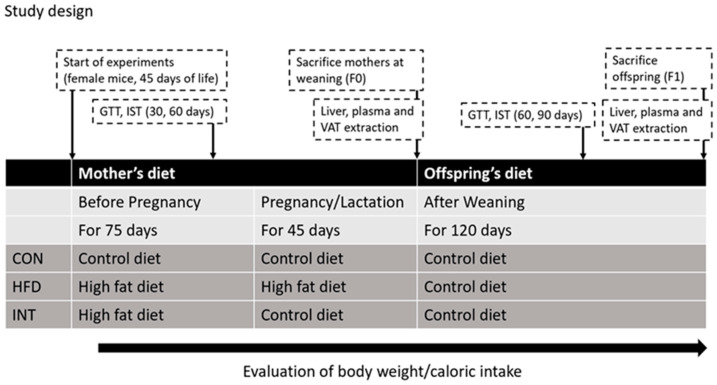
Study design. GTT, glucose tolerance test; IST, insulin sensitivity test; VAT, visceral adipose tissue; F0, mothers, generation 0; F1, offspring, first generation.

For mating, a male was arranged with 2 females in each cage. After mating, females were kept in individual cages, evaluating pregnancy by visual observation until 12 days after mating. After this, the probable date of delivery was estimated. After the 21 days of lactation period, weaning took place and the pups were sexed, grouping them into cages of 5 animals according to sex and to the group of the mother of origin. All pups were kept on a control diet until 120 days old, when they were sacrificed, and tissues of interest were collected and stored until analysis (Figure 2).

For the purposes of this article, only male pups were used, due to the low number of female offspring.

### 2.3. Diets

Certified diets were used, control (Research Diet INC. Rodent Diet with 10% of total calories as fat Product data D12450J. USA), or high-fat (Research Diet INC. Rodent Diet with 60% of total calories as fat. Product data D12492. USA), which supplied enough amounts of fiber, vitamins, and minerals (Supplementary Table S1) and a different fatty acid profile (Supplementary Table S2).

### 2.4. Evaluation of Body Parameters

#### 2.4.1. Measurement of Body Weight and Intake

Food intake and body weight of all mice were assessed and recorded weekly. This assessment started from weaning in the case of the pups, to avoid stressing them during the lactation period.

#### 2.4.2. Weight Measurement of Visceral Fat and Liver

Immediately after euthanasia, the visceral adipose tissue was removed for weighing. The liver was removed, weighed, and immediately frozen in liquid nitrogen to be stored at −80 °C until its use in molecular biology determinations.

### 2.5. Evaluation of Metabolic Parameters

#### 2.5.1. Glucose Tolerance Test and Insulin Sensitivity Test

The glucose tolerance test and the insulin sensitivity test were performed in females prior to pregnancy, the first and second month after starting the experiment with control diet and HFD, as well as in the second and fourth month of the pups’ lives. Both tests were performed 1 week apart, under fasting conditions of at least 6 h, with the animals arranged in individual boxes and water ad libitum. One hour before the start of the test, the dose of glucose or insulin to be injected intraperitoneally was calculated: 1.5 mg per gram of weight (glucose) and 0.75 milliunits of insulin per gram of weight (recombinant human insulin). Glycemia was measured using strips and a glucose monitor, extracting a blood sample from the tail at 0, 15, 30, 60, 90, and 120 min.

#### 2.5.2. Plasma and Tissue Collection

After weaning, the mothers were euthanized. For this, they were first introduced into a chamber with isoflurane vapors added to the physical method of thoracotomy and blood collection, in accordance with the recommendations of the Federation of European Associations for Laboratory Animal Science and the American Association of Veterinary Physicians for rodent euthanasia. After euthanasia, visceral adipose tissue and liver were removed. The liver was kept in liquid nitrogen and stored at −80 °C until it was used for biochemical evaluations of real-time RT-PCR, Western blot, and determinations of total cholesterol and hepatic triglycerides. Additionally, blood samples were obtained from all mothers to measure insulin, leptin, adiponectin, and corticosterone, as well as to evaluate total cholesterol and plasma triglycerides. Samples were obtained from the abdominal aortic artery (0.5–1 mL) in Vacutainer^®^ tubes with EDTA at 0–4 °C, and later centrifuged at 3000× *g* in an Eppendorf centrifuge for 15 min at 5 °C. The supernatant, corresponding to blood plasma, was carefully collected (0.3 mL–0.7 mL) and stored at −80 °C until analysis. These same procedures were also performed on the adult offspring.

#### 2.5.3. Determination of Plasmatic Hormone Levels

Circulating plasma levels of insulin, corticosterone, adiponectin, and leptin were determined by ELISA using specific commercial kits:

- Corticosterone was evaluated with the ELISA kit from the Cayman Chemical Com-pany, MI, USA, No. 501320.
- Insulin with ELISA kit EMD Millipore Corporation, MI, USA, Cat. # EZRMI-13K.
- Adiponectin with ELISA kit EMD Millipore Corporation, MI, USA, Cat. # EZMADP-60K.
- Leptin with EMD ELISA kit Millipore Corporation, MI, USA, Cat. # EZML-82K.

All samples were worked with in duplicate, according to the manufacturer’s instructions.

#### 2.5.4. Determination of Plasmatic and Hepatic Lipids

Quantitative extraction of total lipids from liver samples was carried out according to the Folch method using 100 mg of tissue from the different experimental groups. Liver samples were homogenized in ice-cold chloroform/methanol (2:1 *v*/*v*) in an Ultraturrax homogenizer. Then, magnesium chloride (MgCl2) 0.5N and BHT as antioxidant) was added at a concentration of 0.01% (*w*/*v*). The homogenate was centrifuged at 1500 rpm for 10 min at 4 degrees C. Subsequently, the chloroform (fraction in which lipids are found) was extracted. The final chloroform solution of lipids was dried under a stream of nitrogen and subsequently suspended in PBS 5% Triton X-100 solution. An aliquot of this solution was destined for the determination of hepatic triglycerides using a commercial enzymatic colorimetric kit (Cayman Chemical Company, Ann Arbor, Michigan, USA, N°10010303), following the manufacturer’s instructions. As standard, a triglyceride curve was used, of known concentrations (0; 3.125; 6.25; 12.5; 25; 50; 100 and 200 mg/dL). A second aliquot was used for the determination of plasma triglycerides, using the same commercial enzymatic colorimetric kit applied in plasma. Plasma cholesterol was determined using a commercial enzymatic colorimetric kit (BioSystems S.A., Barcelona, Spain, COD 21505). For the determination of liver cholesterol, a commercial enzymatic colorimetric kit (BioVision, Milpitas Blvd., Milpitas, CA 95035 USA, Catalog # K603-100) was used.

#### 2.5.5. Determination of Hepatic Fatty Acid Profile

##### Extraction of Fat

The total lipid content present in the liver was obtained qualitatively using the modified Bligh and Dyer technique (Bligh and Dyer, 1959). Briefly, 200 mg of liver tissue was homogenized in the cold, after which chloroform and methanol were added in a 2:1 *v*/*v* ratio, along with BHT. In order to provide the solution with a greater ionic strength and thus obtain a better separation of the organic and aqueous phases, 0.5 N MgCl_2_ was added, and the samples were centrifuged at 1500× *g* for 5 min at 4 °C, after which the organic phase (chloroform) was recovered. This cycle was repeated 3 times. Finally, the samples were concentrated by the evaporation of chloroform with nitrogen and then stored at −20 °C.

##### Preparation of Fatty Acid Methyl Esters

Fatty acids obtained from liver tissue were transformed to fatty acid methyl esters (Firestone, 1997) through incubation at 95 °C with 12% BF3 in methanol and NaOH in methanol. Saturated NaOH was then added, after which hexane was added to solubilize the methyl esters. The samples were stored at −20 °C until they were used for analysis via gas chromatography.

##### Analysis of Fatty Acid Methyl Esters

An analysis of the profile of saturated, monounsaturated, and polyunsaturated fatty acids (SFA) methyl esters present in the samples, as well as the relationship between ω-6 and ω-3 fatty acids, obtained using gas–liquid chromatography coupled to ionization is called as a detection method. The equipment used was a Hewlett-Packard, model 6890A, coupled to a capillary column (Agilent HP-88, 0.25 mm × 60 m, 0.2 μm), using hydrogen as the mobile phase. The temperature was programmed from 160 to 220 °C with a temperature ramp of 3 °C/min, reaching a final time of 30 min per injected sample. The detector and injector temperature was 250 °C. Sample fatty acids were identified by comparing their retention times with those of previously injected individual fatty acid standards (Nu-Check Prep., Elysian, MN, USA), via computerized integration (ChemStation Rev A.10.01 (1635), Agilent Technologies, 2003). The internal standard used was C23:0. Results were expressed as grams of fatty acid/100 g EMAG.

### 2.6. Determination of Gene Expression

#### 2.6.1. Total RNA Extraction

Livers stored at −80 °C were pulverized cold, in order to obtain a homogeneous sample for total RNA extraction. Subsequently, the tissue was homogenized using 1 mL of TRIZOL reagent (Invitrogen, Carlsbad, CA, USA) for every 100 mg of tissue and incubated at room temperature for 5 min, according to the manufacturer’s instructions. A total volume of 200 µL of RNAse-free chloroform was added, shaken for 15 s, and incubated again at room temperature for 10 min. The samples were centrifuged at 12,000× *g* and 4 °C for 15 min to later transfer the supernatant to 1.5 mL tubes, to which 500 µL of RNAse-free isopropanol was added, with shaking for 10 s. Then, the tubes were kept at room temperature for 10 min and centrifuged for 8 min at 12,000× *g* and 4 °C in order to precipitate the RNA. Subsequently, the samples were washed with 1 mL of cold RNAse-free ethanol (75% *v*/*v*), shaken, and centrifuged at 7500× *g* for 5 min. The resulting pellet was re-suspended in 40 µL of distilled water treated with DEPC (diethyl pyrocarbonate) and stored at −80 °C until use.

In order to validate the integrity of the extracted RNA, agarose gel electrophoresis was used at a concentration of 1.2% (*w*/*v*) and observed under ultraviolet light. The RNA concentration was determined by absorbance via UV spectrophotometry, at a wavelength of 260 nm, making 1:100 dilutions with distilled water treated with DEPC.

#### 2.6.2. cDNA Synthesis

In 0.2 mL tubes, the following were added: 1µL of oligo dT (deoxythymidine), 1 µg/µL of extracted RNA, and 13 µL of nuclease-free water. These were incubated at 70 °C for 5 min, and then at 4 °C for 5 min. Then, 2.5 µL of nuclease-free water, 5 µL of 5× buffer, 1.5 µL of 10 mM dNTPs (dinucleotide triphosphate), and 1 µL of the reverse transcriptase enzyme M-MLV-RT were added (Moloney Murine Leukemia Virus Reverse Transcriptase; Promega Corporation, Madison, WI, USA), according to the manufacturer’s instructions. The total volume of 25 µL was incubated at 42 °C for 90 min, 70 °C for 15 min, and at 4 °C for 5 min. The concentration of the cDNA obtained was determined via UV spectrometry at a wavelength of 260 nm.

#### 2.6.3. qPCR in Real Time

Gene expression of the CB1 receptor (RCB1), of the endocannabinoid-degrading enzymes FAAH and MAGL, and of the lipogenic genes ACC1, FAS, and SREBP-1c in liver tissue were determined to indirectly assess RCB1 activity. To perform this, cDNA was first synthesized from 2 µL of already diluted RNA using TaqMan^®^ Small RNA Assays, according to the manufacturer’s instructions. Subsequently, the cDNA was quantified in a 1:100 dilution, measuring absorbance at 260 nm.

cDNAs samples were amplified in a LightCycler 2.0 thermal cycler (Roche, Ludwigsburg, Germany), using the Kapa SYBR^®^ FAST qPCR Kit Master Mix (2) Universal (Boston, MS, USA), to observe the amplification products, according to the manufacturer’s specifications. For this purpose, a mixture was prepared containing 3 µL of nuclease-free water, 0.5 µL of each primer at a concentration of 10 µM (forward and reverse), and 5 µL of Master Mix (2), for each sample, and 1.5 µL of cDNA (300 ng/µL). A negative control was used, and as an internal control, the mouse β-actin gene was amplified [16]. From the synthesized cDNA, 1 µL was used for TaqMan MGB qPCR, in duplicate (Ilumina equipment). Table 1 provides the details of the primers used.

### 2.7. Determination of the Amount of Total Protein

#### 2.7.1. Protein Extraction and Quantification

- Protein precipitation

Samples were prepared using 100 mg of pulverized liver tissue, to which 500 µL of RIPA buffer (radioimmunoprecipitation assay; Pierce Biotechnology, Rockford, IL, USA) was added in the presence of protease inhibitors. The samples were shaken for 60 s and kept on ice for 45 min to be homogenized with a polytron (2 times for 15 s). Then, the samples were centrifuged at 14,000× *g* for 10 min at 4 °C and stored at −80 °C until further analysis.

The protein concentration of the supernatant was determined using a commercial colorimetric kit (Bio-Rad, Hercules, CA, USA), via spectrophotometry at a wavelength of 630 nm, with bovine serum albumin as standard protein. Subsequently, they were subjected to 10% SDS-PAGE gel electrophoresis. Protein electrotransfer to PVDF (polyvinylidene fluoride) membranes was performed at constant amperage (60 mA) overnight with continuous stirring at 4 °C.

#### 2.7.2. Western Blot Analysis

To assess the amount of RCB1 and FAAH and MAGL degradation enzymes:

- Incubation with antibodies

Once the electroblotting was finished, the PVDF membranes were stained with a Ponceau Red solution and blocked using the TBS-Tween-milk 5% (*w*/*v*) blocking solution at room temperature for 1 h with gentle shaking. Subsequently, the membranes were washed 4 times for 10 min under strong shaking with TBS-Tween, to be incubated for 2 h at room temperature with the respective primary antibody, and then washed again and incubated with the secondary antibody for 1 h. Finally, the membranes were washed 3 times for 10 min under strong shaking with TBS-Tween and 2 times for 5 min under strong shaking with 1x TBS.

The antibodies used for the indicated proteins were:

- Anti-FAAH antibody (CaymanChem Co., Ann Arbor, Michigan, USA), developed in rabbit, used with a dilution of 1:500.
- Anti-MAGL antibody (CaymanChem Co., Michigan, USA), developed in rabbit, at a concentration of 5 µg/mL, using a 1:120 dilution.
- The peroxidase-conjugated anti-rabbit secondary antibody (Rockland Immunochemicals, Gilbertsville, PA, USA), at a concentration of 2 mg/mL, was used at a dilution of 1:5000.

As a negative control, its corresponding blocking peptide was used, with a blocking peptide-to-antibody ratio of 1:1 (*v*/*v*) (CaymanChem Co., MI, USA). In contrast, as loading control, the determination of β-actin was used with an antibody developed in mice, and a secondary anti-mouse Igg antibody conjugated with peroxidase (Rockland Immunochemicals, Gilbertsville, PA, USA).

- Revealed

The development of the Western blot analysis was performed via chemiluminescence, using the commercial Western Lightning Plus-ECL Enhanced Chemiluminescence Substrate kit (Perkin Elmer, Waltham, MA, USA). In order to obtain the relative quantification, β-actin was used as control load.

### 2.8. Statistical Analysis

In order to establish the sample size, the Primer of Biostatistics: The Program 3.02 program, Stanton A. Glantz, was used, through which it was determined, with 90% power and a 0.05 alpha error, that the appropriate number of animals to be used for each experimental group was between 7 and 9 animals per experimental group, and between 4 and 6 pups per female, for all the parameters evaluated in the present study. These values were obtained by analyzing previously reported data [19,25,26].

Results were expressed as the mean and its corresponding standard deviation (SD) or standard error of the mean (SEM).

Shapiro–Wilk’s tests were previously performed to assess the normality of data distribution and the homogeneity of variance. If the data were normally distributed, parametric statistics were used and, otherwise, non-parametric statistics were used.

To evaluate statistical differences between the different groups on body parameters, hormonal levels, plasmatic and hepatic lipids, hepatic fatty acid profile, gene expression, and amount of total protein, either Mann–Whitney test or Student’s *t* test was used to assess differences between two independent samples. For 3 or more groups of independent samples, either a one-way analysis of variance (ANOVA) or Kruskal–Wallis was applied. For post-hoc testing, Dunn’s multiple comparison was used. GraphPad Prism software (version 9.00 for Windows, GraphPad Software, San Diego, CA, USA, www.graphpad.com, 12 January 2023) was used for the statistical analysis, with a significance level of 95% and a *p* value ≤ 0.05.

## 3. Results

### 3.1. Hepatic Fatty Acid Profile

Table 2 shows the composition of fatty acids in the liver of the progenitors after lactation. There were no differences between groups for palmitic acid, oleic acid, eicosapentaenoic acid, SFA, and MUFA. However, for total stearic acid, linoleic acid, PUFA, LCPUFA and n-6 LCPUFA, DHA, and n-3 LCPUFA, there are significant differences between the control group and HFD, evidencing an increase in response to diet composition. For alpha-linolenic acid, arachidonic acid, and total n-6 LCPUFA, there were significant differences between the HFD groups and the intervention group.

### 3.2. Body, Hormonal, Biochemical, and Metabolic Parameters in Offspring

The final body weight, liver weight, visceral fat weight, and cumulative energy intake at 120 days of life in the offspring, after maintaining a control diet after weaning, are shown in Table 3. There were no significant differences in body weight among the mice descended from control mothers (CON-F1), high-fat diet group (HFD-F1), and intervention group (INT-F1). However, regarding the weight of the liver, a significantly lower weight (20% less) was observed in the adult pups descended from mothers fed with HFD before and during pregnancy and lactation, compared to the other groups. There were no differences in the epididymal fat content, nor in the cumulative energy intake, even though the latter was higher in the INF-F1 group.

**Figure 3:**
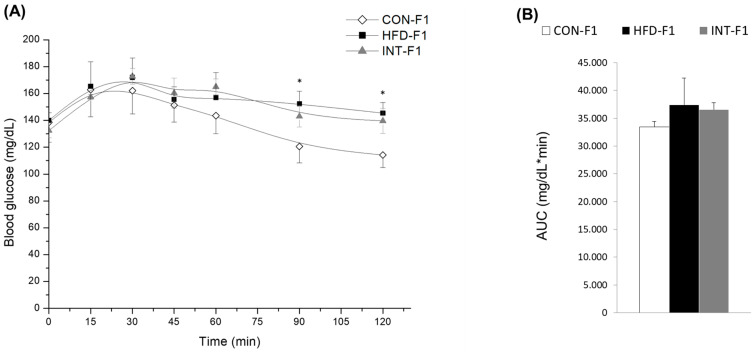

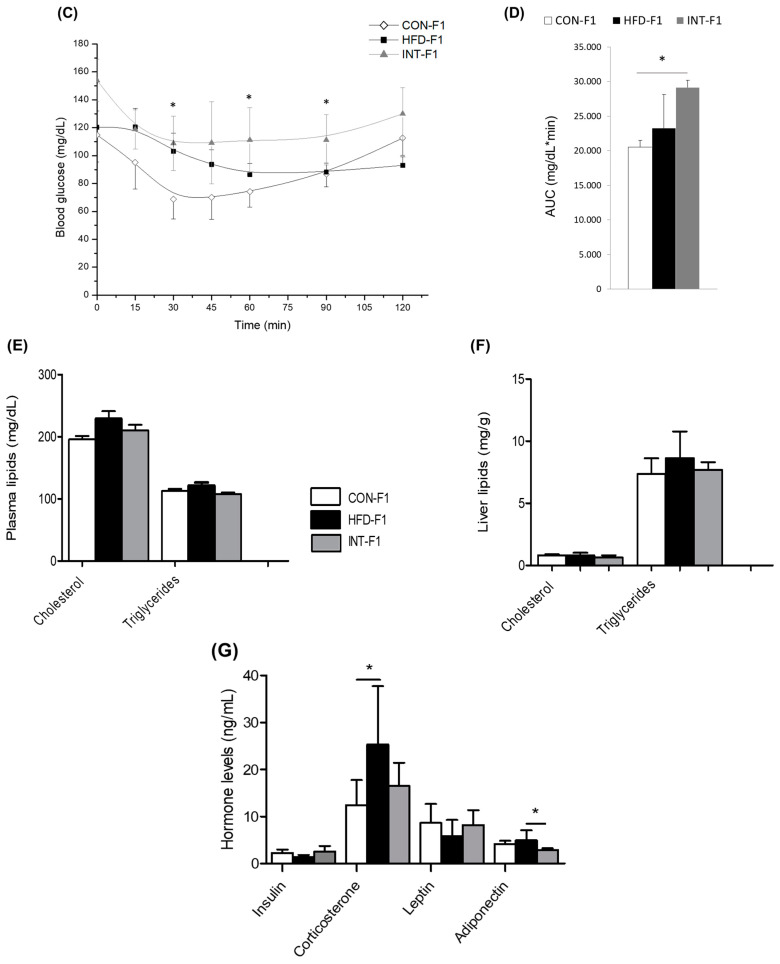
Intraperitoneal (ip) glucose tolerance (**A**) and insulin sensitivity test (**C**) and their respective areas under the curve (AUC) of each test (**B**,**D**) in male pups at 60 days of life according to the type of diet of mothers (n = 6 CON group; n = 5 HFD group; n = 6 INT group). Plasma lipids (**E**), liver lipids (**F**), and plasmatic hormone levels (**G**) in pups according to type of diet of the mothers (n = 5 CON-F1 group, n = 5 HFD-F1 group, and n = 5 INT-F1 group). All pups were fed a control diet during the 120 days of life after weaning. Data presented as mean ± SEM, analyzed with Kruskal–Wallis test, Dunn’s post-hoc, * *p* < 0.05.

Glucose metabolism was also evaluated in the offspring through glucose tolerance test and insulin sensitivity test. Significant differences were observed only at 90 and 120 min in the glucose tolerance test between the F1 control group and the F1 HFD, with no differences in the area under the curve. On the other hand, in the insulin sensitivity test, it is possible to observe significant differences at 30, 60, and 90 min, as well as in the area under the curve between the offspring of the control group and the intervened group, with the latter being greater (Figure 3A–D).

For the levels of plasmatic and hepatic lipids, no differences are observed between the different groups of offspring (Figure 3E,F).

Plasma hormone levels in offspring (Figure 3G) show a significant difference between groups for corticosterone, with mice from HFD mothers having the highest values. The same is observed for adiponectin, where the lowest values are observed in the group of mice from obese mothers treated with a control diet during pregnancy and lactation (INT-F1).

### 3.3. Gene Expression of mRNA of Components of the Endocannabinoid System and Target Genes of the CB1 Receptor in Liver Tissue of Offspring

The mRNA relative expression of the most relevant components of the endocannabinoid system, CB1 receptor and endocannabinoid degradation enzymes, FAAH and MAGL, was evaluated, as well as the expression of genes regulated by this receptor and related to de novo lipogenesis in liver, the factor SREBP-1c lipogenic transcription agent, and its target genes, the enzymes acetyl-CoA-carboxylase 1 (ACC1) and fatty acid synthetase (FAS) in the liver of adult F1 mice, according to type of maternal diet.

**Figure 4:**
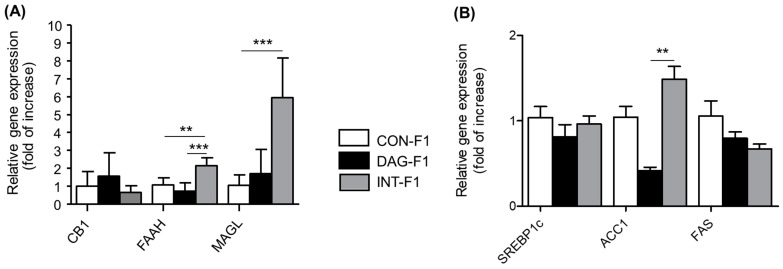
Hepatic gene expression of ECS components (**A**) and RCB1 target genes (**B**) in F1 (offspring) according to the mother’s diet (n = 5 CON group, n = 5 HFD group, and n = 5 INT group). Cq values were normalized by the geometric mean of reference target (β-actin gene). Changes are shown in relation to the values in the control group. Data are presented as mean ± SEM, analyzed with Kruskal–Wallis, Dunn’s post-hoc. ** *p* = 0.0015, *** *p* < 0.0001.

In this regard, it is observed that the adult offspring of the group of obese mothers treated with a control diet during pregnancy and lactation (INT-F1) have a higher hepatic expression of FAAH and MAGL enzyme genes (Figure 4A). ACC1 mRNA expression is also upregulated in the INT-F1 group (Figure 4B). In contrast, no differences were observed in gene expression of SREBP-1c and FAS.

### 3.4. Western Blot Analysis of Components of the Endocannabinoid System in Liver Tissue in Offspring

**Figure 5:**
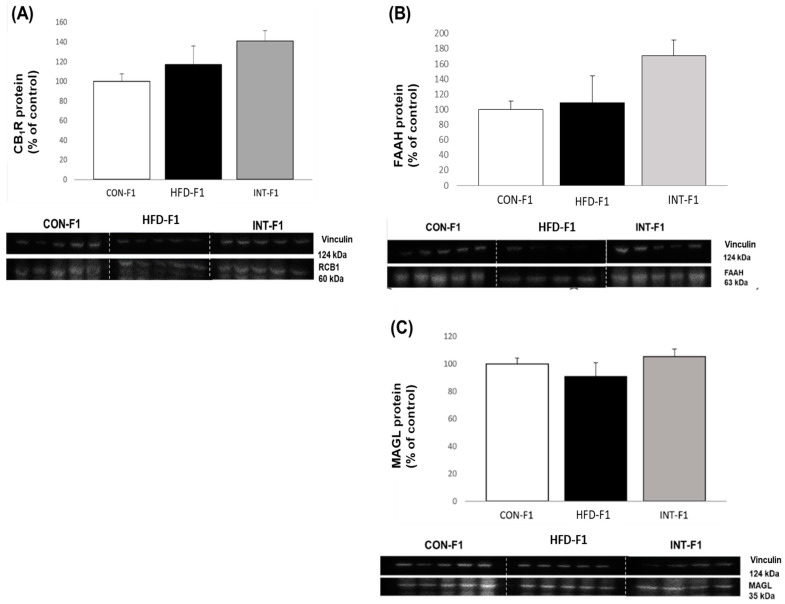
Protein levels of CB1 receptor (**A**); FAAH (**B**); MAGL (**C**) in adult pups (n = 5 per group). All pups were fed a control diet during their 120 days of life. Data are presented as mean ± SEM, analyzed with Kruskal–Wallis test and Dunn’s post-hoc.

Figure 5 shows the level of the CB1R protein, as well as the FAAH and MAGL enzymes in F1, in 120-day-old mice fed with control diet since weaning. Band quantification was normalized in relation to vinculin expression. There is a trend of higher protein levels in the INT-F1 group; however, no statistic differences were found for any of the proteins measured.

## 4. Discussion

An intervention model was generated to study the effect on the offspring of a control diet during pregnancy and lactation of obese progenitors C57BL/6 mice. The INT-F1 group showed a greater liver weight compared to the HFD-F1 group and a lower sensitivity to insulin, compared to the control group. Regarding hormonal parameters, a normalization of adiponectin and corticosterone levels was observed in the intervened group, with values comparable to those of the CON-F1 group. The results also suggest that the diet intervention in obese progenitors increased the hepatic gene expression for enzymes catalyzing the degradation of the main endocannabinoids in the offspring, FAAH and MAGL, compared to the other two groups of animals (control and HFD). The same occurred with the expression of ACC1, a target gene of the CB1 receptor. However, these differences were not observed at protein levels.

Several studies have shown that a high energy intake, based on fat (palmitic acid and oleic acid, in particular) during pregnancy can generate metabolic alterations of lipotoxic origin, affecting the offspring, which is known as fetal programming [27,28,29,30]. Maternal HFD models have shown long-term metabolic effects, including an association with higher levels of body fat, body weight, leptin, glucose, insulin, and triglycerides in the offspring, even though sources of variation have been observed between the studies, related to the murine model and the type of high-fat diet [31] as well as gender-associated differences in pups receiving a control diet after weaning [32]. In this study, maternal exposure to HFD did not affect the final body weight of the offspring in adulthood (120 postnatal days), adiposity, plasmatic or hepatic lipids, nor glucose tolerance. Some of these results are not consistent with those reported by other studies where the effect of maternal HFD has been evaluated. In this regard, a meta-analysis carried out by Ribaroff et al. [33] showed that maternal exposure to HFD during pregnancy and lactation increased final body weight, adiposity, triglyceridemia, cholesterolemia, and insulinemia in both pup genders (females and males), although hyperglycemia was only observed in female pups. This is consistent with our results, since we did not observe differences in glycemia between the HFD-F1 group and the CON-F1 group. Regarding insulin sensitivity, no differences were observed either. This result is controversial since exposure to a HFD was reported in one study to promote a decreased insulin sensitivity in mice even in the second generation [34], and in another study, it was observed that this HFD exposure during lactation was associated with hyperinsulinemia in the offspring at 90 days [35], which we did not observe in the HFD-F1 group.

On the other hand, the INT-F1 group showed a lower insulin sensitivity at 60 days postnatal, with a greater AUC in the test, compared to the CON-F1 group, even when no significant changes were observed in the glucose tolerance test. An explanation for these results regarding insulin sensitivity in the offspring could be related to the adiponectin levels observed in the INT-F1 group, which were significantly lower, compared to the HFD-F1 group. The biological effect of adiponectin has been described to increase insulin sensitivity through an increase in fatty acid oxidation and inhibition of hepatic glucose production [36].

In relation to the components of the ECS, the hepatic expression of FAAH and MAGL, as well as the expression of ACC1, increased in the INT-F1 group. However, there were no changes at protein levels, and although there is a tendency toward an increase in the evaluated ECS components, these do not show statistically significant differences and protein activity was not measured. The results in ACC1 are consistent with the findings of the study by Wesolowski et al. [37], which reported an increase in the levels of hepatic expression of ACC1 after using a control diet during pregnancy in non-human primates, possibly indicating a greater fatty acids synthesis. To our knowledge, there are no other published studies evaluating the effect of a normalization diet on the components of the liver ECS, which makes a more extensive comparison of our results impossible.

No large effects of HFD were found on the offspring except for adiponectin and corticosterone levels. The exposure to HFD during pregnancy and lactation significantly increased the levels of adiponectin, in comparison to the control group. Similar results were reported in a study conducted in rats where mothers were fed a HFD during pregnancy and lactation and plasma adiponectin levels were altered: they reduced in female pups and increased in males [38]. On the other hand, the relationship between the hyperactivity of glucocorticoid metabolism and the pathophysiology of obesity and its complications has been postulated in different studies [39,40,41]. Prenatal stress can produce hormonal changes that can last until adulthood, where the hypothalamic–pituitary–adrenal (HPA) axis plays a fundamental role [42]. It has been previously described that HFD during pregnancy in rats leads to HPA axis programming in the first generation of male offspring. Our results show high levels of corticosterone in the HFD-F1 group, suggesting an exacerbated response of the HPA axis. This is consistent with another study showing that mice subjected to stress during lactation have a significant increase in HPA axis hormones, ACTH and corticosterone, both at 21 and 135 days, together with overweight in adulthood, compared to control mice [26].

Negative feedback between glucocorticoids and the ECS has been described [43]. In this regard, it is necessary to analyze the modulation exerted by the endocannabinoid system on the activity of the HPA axis. Initial studies evaluating the action of endocannabinoids, such as anandamide, on the HPA axis reported that intracranial administration of EC induced a dose-dependent increase in ACTH and corticosterone levels [44]. However, subsequent research demonstrated that endocannabinoids negatively modulate HPA axis activity, reinforcing the idea that there is an EC tone that exerts inhibitory actions on the axis activation [19,45]. D’Asti et al. [46] showed that the exposure to a diet rich in n-6 fat (HF-n-6) or rich in n-3 (HF-n-3) to pregnant mice during the last week of gestation and throughout the lactation period increased the corticosterone levels in the offspring (postnatal 10 days).

Other studies have evaluated the effects of HFD and SEC in the offspring’s brain, adipose tissue and/or liver. In this regard, we found that the expression levels of FAAH are lower for HFD-F1 compared to the INT-F1 group. Osei-Hyiaman et al. [16] previously reported a decreased expression of these endocannabinoid-degrading enzymes after chronic exposure to a high-fat diet.

It is interesting that in the progenitors exposed to HFD, the hepatic fatty acid profile evaluated after pregnancy and lactation period showed docosahexaenoic acid (DHA), total polyunsaturated fatty acids (PUFA), and total long-chain acids of the n-3 family (n-3 LCPUFA) values doubling the levels of the progenitors with the control diet. An explanation for this finding could be the fatty acid composition of the high-fat diet used in this study, containing amounts of α-linolenic acid three times higher than the control diet, and linoleic acid five times higher, in the absence of DHA (data in Supplementary Materials). It is known that PUFA levels in the body are influenced by dietary factors such as the intake of essential fatty acids, as well as by the enzymatic activity of desaturases and elongases [47,48]. The consumption of α-linolenic acid through the diet, as a precursor of DHA, matters as much as its relationship with the intake of linoleic acid, influencing the rates of synthesis of polyunsaturated fatty acids of the organism. If the diet has a low content of α-linolenic acid, it leads to a decreased DHA synthesis [49,50]. On the other hand, the hepatic synthesis of AA and DHA is a necessary metabolic process to ensure a constant supply of these LCPUFA to other tissues [51] and in women; moreover, it is more efficient than in men due to the positive active control of estrogens on the activity of desaturases [52]. Women may also store LCPUFA in pregnancy and lactation to ensure adequate flux of AA and DHA to the fetus and newborn [53]. During pregnancy, AA and DHA transport from the mother to the fetus is facilitated by specific transporter proteins, enhancing the transfer of these fatty acids across the placenta [54]. Regarding maternal hepatic fatty acids profile, it was observed that dietary intervention (INT-F0) normalized its values, which increased with HFD. There are no previous studies with a similar design, changing to a normocaloric diet after pregnancy and lactation in progenitors with diet-induced obesity. However, the study by Kowalski et al. [55] reported that switching from an HFD in obese mice to a low-calorie diet reduced energy intake, resulting in reductions in fat mass and liver triacylglycerol and diacylglycerol levels, similar to our results.

The few effects of the early exposure to HFD on the offspring are consistent with what was observed in the mothers, which only showed differences in some of the parameters evaluated. It has been described that the environmental sensitivity of the epigenome is considered an adaptive mechanism by which the developing organism adjusts its metabolic and homeostatic systems to adapt to the expected extra-uterine environment. Inadequate adaptation can produce a poor adjustment that results in a later increased susceptibility to disease [56]. In this study, the offspring always received a control diet regardless of their progenitor’s type of diet, which may have contributed to a few differences in adulthood.

Regarding the limitations of this study, the number of animals in the HFD-F1 group is only five animals, due to the fact that the obese progenitors presented greater difficulties in becoming pregnant and had fewer offsprings. In addition, a mortality of 100% was observed in the female pups of the HFD-F1 group, which is why this work only shows results in male pups. Another study has also reported an unexpected increase in mortality greater than 50% [57]. Although the high-fat diet used was not the most suitable for the development of the negative metabolic effects that were sought, it turned out to be a finding in our study. The lipid composition of the high-fat diet used, with a moderate amount of saturated fat and a high content of ALA, AL, and ARA, as a precursor of endocannabinoids, was not enough to promote the development of the metabolic syndrome in the progenitors, impacting the subsequent expected response on fetal programming and the SEC. In addition, although the expression of the CB1 receptor’s target genes (SREBP1c, ACC1 y FAS) were measured as an indirect way to assess their activity, the quantity of these proteins was not measured, which leaves an information gap that needs to be answered via future research.

One of the strengths of this study is having performed the measurement of the hepatic fatty acid profile, showing us consistency related to the lipid composition of the diets used. The fatty acids profile in the progenitor’s livers decrease in the intervention group (INT-F0), being similar to the values of the control group. Additionally, it should be noted that this is the first study that shows an effect of the use of a diet intervention during pregnancy and lactation on ECS components in the liver of the offspring.

Although it is not possible to extrapolate all the observations made in this animal model to what occurs in humans, the research question that motivated this study is related to the nutritional intervention that is routinely performed in obese women starting pregnancy. These women are usually told to eat a normal-calorie-healthy diet to control gestational weight gain and avoid complications; nevertheless, unhealthy eating habits are frequently resumed after birth. The intrauterine programming that occurs during the gestation period is affected by maternal diet. However, it also needs certain extrauterine conditions to express itself, and if postnatal feeding is consistent with the intrauterine environment, this effect can be developed. In our study, the obese parents were on a high-fat diet for 2.5 months before pregnancy and the intervention group was exposed to the normalization diet for 42 days (the entire period of pregnancy and lactation), and subsequently, the offspring was fed post-weaning with a control diet, independent of the diet of its progenitors. Although this nutritional intervention is desirable to be maintained in women with gestational obesity, it does not occur. Some authors have even suggested that obese women should try to lose weight before pregnancy, and not during pregnancy, considering the possibility that the exposure time to a normocaloric diet may not be enough to prevent the fetal programming caused by maternal obesity.

In summary, our results show that with this intervention model during the pregnancy and lactation in obese progenitor C57BL/6 mice, there is a normalization effect in the male offspring of some of the parameters evaluated, specifically with regard to the previous adiponectin and corticosterone levels, with values comparable to those of the control group, as well as an increase in the hepatic expression of genes for enzymes that degrade the main endocannabinoids in the offspring, FAAH and MAGL, compared to the other two groups of animals, which possibly generates a regulation of the hyperactive ECS in the context of obesity. However, further studies are required to assess the effects of maternal obesity on ECS, as well as the effects of a normalization diet on female offspring. Future work also requires studying other tissues involved in body weight regulation, such as adipose tissue and the hypothalamus. Finally, intervention studies in humans could allow us to validate the relationship between nutritional interventions during pregnancy and lactation, levels of ECS components in normal weight and obese women, and the fetal programming of offspring in these conditions.

## Figures and Tables

**Table 1:** Primers used in real-time qPCR determinations.

Enzyme	Sequence	T
AAC1 Fw qRT-PCR	5′-CCT CCG TCA GCT CAG ATA CA-3′	56.0 °C
ACC1 Rev qRT-PCR	5′-TTT ACT AGG TGC AAG CCA GAC A-3′	56.2 °C
FAS1 Fw qRT-PCR	5′-GGA GGT GGT GAT AGC CGG TAT-3′	58.5 °C
FAS1 Rev qRT-PCR	5′-TGG GTA ATC CAT AGA GCC CAG-3′	56.0 °C
FAS2 Fw qRT-PCR	5′-GGC ACT GAC TGT CTG TTT TCC A-3′	57.5 °C
FAS2 Rev qRT-PCR	5′-GTA AAA ATG ACA CAG TCC AGA CAC TTC-3′	56.0 °C
YWHAZ Fw qRT-PCR (housekeeping)	5′-TTG ATC CCC AAT GCT TCG C-3′	55.9 °C
YWHAZ Rev qRT-PCR (housekeeping)	5′-CAG CAA CCT CGG CCA AGT AA-3′	57.8 °C

**Table 2:** Hepatic fatty acid profile. Data are presented as mean ± SD and expressed as µmol of fatty acid per g of liver, n = 5 animals. Kruskal–Wallis, Dunn’s post-hoc. Statistical significance (*p* < 0.05), different letters indicate significant differences between groups.

	Groups of Study			
	Control-F0	High Fat Diet-F0	Intervention-F0	*p* Value
Fatty Acid	Fatty Acid Composition (FAME)			
C16:0	44.2 ± 15.2	80.2 ± 19.9	51.4 ± 17.1	NS
C18:0	15.4 ± 5.5 ^a^	26.2 ± 2.7 ^b^	17.4 ± 3.8	0.0336
C18:1n-9	112.8 ± 51.1	112.1 ± 23.0	111.9 ± 36.0	NS
C18:2n-6 (LA)	20.8 ± 6.0 ^a^	68.4 ± 19.0 ^b^	22.6 ± 6.6	0.0380
C18:3n-3 (ALA)	0.6 ± 0.2	1.9 ± 0.7 ^b^	0.6 ± 0.3 ^a^	0.0422
C20:4n-6 (AA)	14.1 ± 3.1	23.3 ± 1.9 ^b^	13.0 ± 6.4 ^a^	0.0436
C20:5n-3 (EPA)	0.2 ± 0.06	0.4 ± 0.1	0.3 ± 0.06	NS
C22:6n-3 (DHA)	7.7 ± 1.5 ^a^	15.0 ± 2.3 ^b^	9.1 ± 2.0	0.0380
SFA	63.0 ± 25.0	108.0 ± 22.9	70.3 ± 21.0	NS
MUFA	124.0 ± 55.1	117.0 ± 23.0	133.0 ± 39.3	NS
PUFA	45.7 ± 11.1 ^a^	114.2 ± 25.3 ^b^	48.4 ± 5.7	0.0380
LCPUFA	22.7 ± 1.6 ^a^	39.5 ± 1.4 ^b^	23.2 ± 1.2	0.0363
n-6 LCPUFA	14.8 ± 1.5	24.1 ± 1.2 ^b^	13.8 ± 1.0^a^	0.0423
n-3 LCPUFA	7.9 ± 0.6 ^a^	15.4 ± 0.5 ^b^	9.4 ± 0.04	0.0380
n-6/n-3 LCPUFA ratio	1.9 ± 0.05	1.6 ± 0.04	1.5 ± 0.05	NS

**Table 3:** Body parameters in pups fed with control diet for 120 days of life, obtained at sacrifice. Data are presented as mean ± SEM; Kruskal–Wallis test, Dunn’s post-hoc, statistical significance *p* < 0.05; different letters indicate significant differences between groups.

Variable	Groups of Study			
	CON-F1 (n = 10)	HFD-F1 (n = 5)	INT-F1 (n = 10)	*p* Value
Body weight (g)	32.3 ± 0.8	29.1 ± 0.8	29.9 ± 0.9	0.08
Liver weight (g)	1.2 ± 0.04 ^a^	0.96 ± 0.13 ^b^	1.14 ± 0.04 ^a^	0.05
Visceral fat weight (g)	1.09 ± 0.05	0.84 ± 0.16	0.89 ± 0.07	0.15
Accumulated energy intake (kcal)	4815.9 ± 133.4	4677.3 ± 126.3	5680 ± 52.09	0.09

## Data Availability

Data sharing not applicable. No new data were created or analyzed in this study. Data sharing is not applicable to this article.

## Supplementary Materials

The following supporting information can be downloaded at: https://www.mdpi.com/article/10.3390/nu15163531/s1.
