# Supplementary material for: Effects on Fetal Metabolic Programming and Endocannabinoid System of a Normocaloric Diet during Pregnancy and Lactation of Female Mice with Pregestational Obesity
Source: Nutrients. 2023 Aug 11;15(16):3531. doi: 10.3390/nu15163531 (PMC10458167; doi:10.3390/nu15163531)
Supplement: Supplementary file 1 [file nutrients-15-03531-s001.zip › nutrients-2539272-supplementary.pdf]

Supplementary material

Supplementary Table S1. Nutritional composition of diets.

	Ingredients	Control diet (D12450J)	High fat diet (D12492)
Proteins (% total kcal)	Casein, L-cystine	20	20
Carbohydrates (% total kcal)	Corn starch, sucrose	70	20
Fats (% total kcal)	Soybean oil, lard	10	60
Mineral Mix (g)	Potassium, Calcium, Sodium Chloride, Magnesium, Manganese, Iron, Zinc, Chromium, Copper	50	50
Vitamin mix (g)	Vit. E, niacin, thiamine, biotin, acid. pantothenic, vit. D3, vit. B12, vit. A, pyridoxine, riboflavin, Ac. folic	3	30
Fiber (g)	Cellulose	50	50
Energy (kcal/g)		3.82	5.21

Supplementary Table S2. Diet fatty acid profile.

	Control diet (D12450J) (g/100 g of diet)	High fat diet (D12492) (g/100 g of diet)
Saturated Fatty Acids		
C16:0 Palmitic Acid	0.635	6.471
C18:0 Stearic Acid	0.317	3.671
C20:0 Eicosanoic Acid	0.012	0.067
C22:0 Docosanoic Acid	0.010	0.015
Total saturated fatty acids	1.014	10.654
Monounsaturated fatty acids		
C16:1 Palmitoleic Acid	0.031	0.453
C18:1 Oleic Acid	1.145	11.238
C20:1n9 Eicosaenoic Acid	0.016	0.184
C22:1n9 Erucic Acid	0.000	0.000
Total monounsaturated fatty acids	1.191	11.875
Polyunsaturated Fatty Acids		
C18:2n6 Linoleic Acid	1.476	7.341
C18:3n3 α-Linolenic Acid	0.167	0.543
C20:4n6 Arachidonic Acid (ARA)	0.004	0.067
C20:5n3 Eicosapentaenoic Acid (EPA)	0.000	0.000
C22:6n3 Docosahexaenoic Acid (DHA)	0.000	0.000
Total polyunsaturated fatty acids	1.661	8.253
